# Pernicious Pathogens or Expedient Elements of Inheritance: The Significance of Yeast Prions

**DOI:** 10.1371/journal.ppat.1003992

**Published:** 2014-04-10

**Authors:** James S. Byers, Daniel F. Jarosz

**Affiliations:** Departments of Chemical and Systems Biology and of Developmental Biology, Stanford University School of Medicine, Stanford, California, United States of America; Washington University School of Medicine, United States of America

## Prion Harboring Yeast Strains Found in Nature

Prions are “infectious” misfolded protein states that can template their self-perpetuating conformations onto other molecules of the same type. This unusual folding landscape drives a paradigm-shifting mechanism of inheritance based on changes in protein conformation rather than changes in nucleic acid. The first prion discovered, Prion Protein (PrP), is the causal agent of several human neurodegenerative diseases, including kuru and Creutzfeldt-Jakob [1]. This history has engendered the widespread perception that prions are inherently pathogenic. However, many additional prions have now been found in other eukaryotes in which they influence diverse biological processes and can produce beneficial traits. The most well-characterized of these are found in *Saccharomyces cerevisiae* and other fungi, such as *Podospora anserina* [2].

The best-studied prion is the yeast translation termination factor Sup35. In its soluble form, this protein promotes the faithful termination of protein synthesis. However, in its self-perpetuating prion form, known as [*PSI* ^+^], most Sup35 is sequestered into insoluble amyloid fibers. This increases translational readthrough of stop codons and leads to a variety of phenotypic effects [3]. Most of these traits involve multiple loci and arise from previously cryptic genetic variation (e.g., polymorphisms downstream of stop codons) [3]. That is, [*PSI* ^+^] provides access to genetically complex traits in a single step. Sup35 variants from fungi separated by over 100 million years of evolution retain the ability to acquire [*PSI* ^+^] [4], [5].

In the laboratory, cells spontaneously acquire [*PSI* ^+^] at low frequencies (∼1 in 10^6^) [2], [6]. Some have suggested that this element thus provides a “bet-hedging” mechanism, promoting survival by speeding the manifestation of new heritable traits in fluctuating environments [6]. Any population of cells that grows to an appreciable size will include some [*PSI* ^+^] individuals. Although these [*PSI* ^+^] cells are genetically identical to the majority, they will nonetheless express different traits. Even if the phenotypes produced by [*PSI* ^+^] are neutral or detrimental in many environments, a rare strong selective advantage would ensure survival of the population in conditions in which it would otherwise perish [3]. Indeed, population genetics modeling suggests that even extremely rare selective advantages are sufficient to explain the [*PSI* ^+^] switching rates observed in laboratory growth conditions [7].

This line of thinking is intuitively appealing and could easily be extended to other prions. Yet, an opposing view posits that yeast prions are in fact diseases or even artifacts of laboratory culture. A key line of evidence supporting this view had long been the absence of prions in natural yeast isolates that had been tested [8]. However, the recent acquisition of many additional sequenced wild yeast strains has revealed prions' common presence in nature. Analysis of nearly 700 such strains from diverse ecological niches revealed that many harbored [*PSI* ^+^] and/or other prions [9]. [*PSI* ^+^] was found in ∼2% of the strains. [*MOT3* ^+^], which is formed by the Mot3 transcription factor and provides resistance to cell-wall toxins, was observed in ∼6% of the tested strains [9]. Moreover, one third of the wild strains analyzed had additional phenotypes with the unusual features of prion-based inheritance (e.g., cytoplasmic transmission and strong dependence on the activities of molecular chaperones) [9]. These observations have not eliminated the “prions as diseases” argument (see below), but they clearly demonstrate that prions are not merely an anomaly created in the laboratory. Rather, they play a crucial role in shaping the behavior of natural populations.

## Sophisticated “Bet-Hedging” Devices or Selfish Parasitic Elements?

[*PSI* ^+^] and [*MOT3* ^+^] clearly are not universally beneficial or they would have swept natural yeast populations. Indeed, models comparing [*PSI* ^+^] prevalence with that of other “infectious” elements, such as 2-micron plasmids, suggest that the fitness cost of [*PSI* ^+^] may be ∼1% on average [10], [11]. However, detriment can also be punctuated by periods of strong benefit. Models of [*PSI* ^+^]'s fitness costs rely on a variety of estimated and measured parameters. Key among these are prevalence in nature, rates of outcross mating, rates of gain and loss (spontaneous and induced), population size, and the number of generations between periods of selective benefit [7], [10], [12], [13]. The calculated fitness cost of [*PSI* ^+^] (∼1%) assumes a 1% prevalence in natural populations and a loss rate of 10^−5^ [10]. However, rates of [*PSI* ^+^] loss have not been rigorously examined over a wide variety of strains and circumstances [11], [14]. Strikingly different conclusions, e.g., a benefit of 1%, would arise from calculations using lower published outcross mating frequencies (∼10^−5^) [12], [13] and higher [*PSI* ^+^] loss rates (∼10^−2^), underscoring the need for additional measurements of these parameters. Indeed, there is evidence that prion loss rates can sometimes be very high. [*MOT3* ^+^] is lost at low frequencies under many conditions, but it undergoes uniform reversion during hypoxia [15]. That is, specific environmental conditions completely eliminate the prion state. Also absent from most models of prion benefit and detriment is the observation that [*PSI* ^+^]-dependent traits can be genetically assimilated in a single meiosis [9], [16]. This could lead to the retention of a beneficial trait without a need to retain the prion. Additional measurements and models are critical to assess the adaptive value of prions in natural settings.

## Prions Modulate Natural Genetic Diversity

Prion-based phenotypes often depend strongly on genetic background. For [*PSI* ^+^], this is likely because sequences downstream of stop codons are under low selective pressure and are relatively free to accumulate mutations [9]. For example, one [*PSI* ^+^] wine strain was resistant to both acidic pH and fluconazole. Another [*PSI* ^+^] strain isolated from grapes was instead resistant to DNA-damaging agents. [*MOT3* ^+^] phenotypes, too, depend on variation harbored in the host strain. A [*MOT3* ^+^] strain isolated from holly berries was resistant to the cell-wall toxin calcofluor white, likely reflecting Mot3's transcriptional regulation of genes involved in cell-wall synthesis. However, another [*MOT3* ^+^] strain isolated from Finnish soil was acid resistant. Importantly, all of these traits were prion dependent and were eliminated with transient inhibition of Hsp104, the disaggregase required for prion propagation. In total, roughly half of the phenotypes bestowed by these prions were adaptive, a key distinction from previous studies of both [*PSI* ^+^] in laboratory strains (in which only 25% were adaptive) [17] and random mutations, which are heavily biased toward neutral and detrimental fitness effects [18].

Releasing the phenotypic output of silent genetic variation is only one of the mechanisms by which prions elicit phenotypic diversity. Many prions themselves adopt multiple self-perpetuating conformations, each of which drives unique phenotypes. This is vividly apparent for [*PSI* ^+^], in which differences in the physical characteristics of amyloid fibers correlate with the strength of the prion phenotype (for example, strong, weak, branching, etc.) [19]. These prion “strains” are distinct and stable. Moreover, introduction of one conformation leads to the exclusive propagation of that “strain” and its corresponding phenotype. For example, [*PSI* ^+^] “strains” vary in their levels of stop codon suppression as well as their levels of toxicity [17]. Indeed, the ensembles of [*PSI* ^+^] conformations maintained in vivo and present in wild strains are likely biased to exclude those that might be strongly toxic (for further information, see [17]). The extent to which different prions adopt “strains” also differs extensively and depends strongly on environmental conditions [19].

At least one prion, [*Het-s*] from *P. anserina* [20], does not appear to form “strains”. Given the fundamental and stereotyped role of Het-s in *P. anserina's* life cycle (regulating heterokaryon compatibility and nonsexual mating), it is perhaps not surprising that this prion's folding landscape would be more highly regulated than other prion states. Indeed, this observation has been cited by those who view prions as beneficial and those who view them as a disease state alike. The presence of “strains” could reflect a lack of selection on the prion state [21], but it is equally appealing to posit that an ensemble of distinct prion strains would provide additional layers of phenotypic fine-tuning to fuel “bet-hedging” strategies and enable survival [22].

## Prions Are Environmentally Responsive

**Figure 1:**
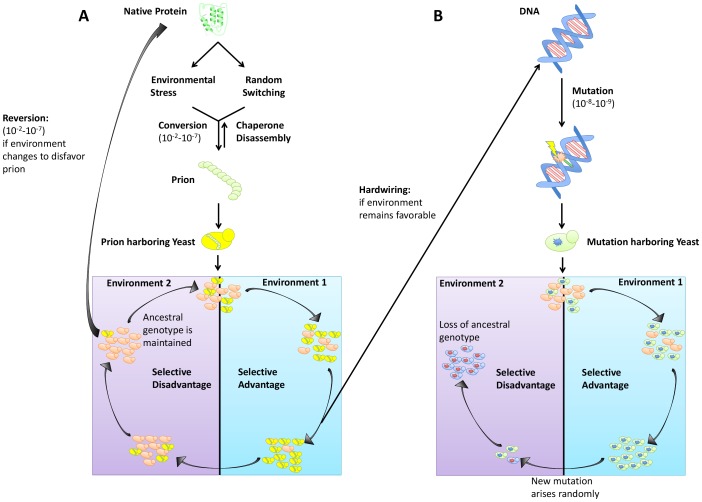
Trade-offs between prion switching and genetic mutation. (A) Prion switching can occur at a rate between 10^−2^ and 10^−7^ per generation and is strongly influenced by environmental stress. Yeast harboring a prion are genetically identical to the original population but can have a selective advantage in certain environments. If the environment remains favorable, traits bestowed by the [*PRION* ^+^] state can be hardwired into the genome via genetic assimilation. However, if the environment changes to disfavor the prion, reversion to the [*prion* ^−^] state can occur. (B) Genetic mutation occurs at a lower frequency (10^−8^–10^−9^ per generation) than prion switching. An allele granting a selective advantage will sweep a population. However, if that allele becomes disfavored, selection of a new mutant will occur, and the original genotype will be lost.

In addition to creating phenotypic diversity in established ecological niches, prions provide a mechanism for rapid adaptation during periods of environmental fluctuation and stress. It is textbook knowledge that genetic mutations can lead to adaptive phenotypes (although most are neutral or detrimental). However, evolution by mutation can come at the cost of “stranding” a population in a maladaptive state should the environment again change (Figure 1B). In contrast, prion-based inheritance circumvents this evolutionary “lock-in” (Figure 1A). Extreme reliance on protein homeostatic machinery also intrinsically links prion induction to environmental stress, providing a mechanism by which organisms can heritably diversify their phenotypes precisely when they are poorly adapted to their environments. Strong increases in the rate of [*PSI* ^+^] formation have been observed for Sup35 mutants in a wide variety of toxic conditions [23]. The mechanistic details of this mechanism remain to be established (four of these conditions did not increase prion switching of wild-type Sup35 [10]). Importantly, however, this phenomenon would nonetheless appear to reflect cells truly “hedging their bets” for cases in which it has been observed: switching correlates with the severity of the stress, regardless of whether [*PSI* ^+^] will provide any benefit to the population.

The effect of environmental stress on prion induction can also be highly specific. For example, [*MOT3* ^+^] acts as an environmentally responsive “switch” to regulate multicellularity between glucose fermentation and ethanol respiration. It is induced in response to ethanol stress (∼10-fold) and subsequently lost in hypoxic conditions [15]. Likewise, the [*MOD* ^+^] prion, which provides resistance to antifungal drugs, is specifically induced by those same agents [24], offering a powerful example of how prions can drive adaptation in fluctuating environments and bear many features of Lamarckian evolution [25].

## Loss-of-Function versus Gain-of-Function

Most naturally arising mutations are neutral or loss-of-function mutations. In some cases, even these mutations can be adaptive (if the energy cost associated with maintaining a gene outweighs its benefits in the current environment [26], [27]). Examining prions through the lens of [*PSI* ^+^] would suggest that prion-mediated aggregation involves loss of a protein's normal cellular activity upon sequestration and/or toxicity of the aggregate. Indeed, the [*URE3*] prion, formed by the nitrogen catabolite repressor Ure2, likewise mimics loss of Ure2 function. For many years, this paradigm dominated thinking in the field with a key exception: [*Het-s*] from *P. anserina*, in which aggregation prevents fusion between cells of opposite mating-type loci [20]. In the [*prion* ^−^] state, cells of opposite mating type (*het-s* and *het-S*) undergo hyphal anastomosis and facilitate spreading of harmful plasmids and fungal viruses [28]. Recent structural work has demonstrated that this [*Het-s*] gain of function arises from exposure of a domain that targets het-s to the membrane [28].

Indeed, other prions produce adaptive gain-of-function phenotypes. For example, unlike *mot3* deletion, [*MOT3* ^+^] causes colony flocculation at the end of fermentation. Another prion that displays gain-of-function phenotypes is [*GAR* ^+^], which allows cells to overcome glucose repression [20]. Propagation of this element from one generation to the next depends upon a multiprotein complex. However, deletion of the component proteins does not relieve glucose repression [29], suggesting this function is gained in the [*PRION* ^+^] state. Finally, adaptive gain-of-function mechanisms have also been found in potential prion proteins from higher organisms. As just one example, the RNA-binding protein CPEB promotes long-term memory formation in its amyloid-like oligomeric state at the synapses in both *Drosophila* and *Aplysia* [30].

## The Trade Off: Prions versus Genetic Mutation

Perpetual cycling in temperature, humidity, and nutrient availability are realities of microbial life. This constant state of environmental flux means that success requires adaptability. One advantage prions have over genetic mutation in establishing diversity is the rate of prion induction versus the rate of spontaneous mutation. Whereas genetic mutations typically occur spontaneously at a rate between 10^−6^ and 10^−8^ (Figure 1B), prion switching can occur much more frequently (between 10^−2^ and 10^−7^) (Figure 1A) [2], [6]. Not only does this allow for faster adaptation during times of environmental stress, it also creates more heterogeneity in a population without a corresponding increase in population size. Reversion of the prion state is also more facile than reversion of mutation, providing a complementary survival advantage should the environment again change to favor the [*prion* ^−^] state (Figure 1A).

The question that remains is this: would nature support a system in which the effects are often detrimental in order to serve as a high-priced insurance policy against unpredictable future stressors? The [*MOD* ^+^] prion showcases the “give-and-take” of this compromise. Although it impairs growth in nutrient-rich conditions, this prion safeguards the population upon exposure to antifungal agents [24]. This fitness landscape mirrors the evolution of antibiotic resistance via mutation, in which adaptive variants often manifest with fitness costs in other conditions [31].

Population genetics predicts that “bet-hedging” mechanisms will provide a strong advantage over mutation on long time scales in irregular selective landscapes [7], [32]. Calculations using established measurements of population size, structure, and environmental fluctuations suggest that the benefits of prion-based “bet-hedging” outweigh its costs, motivating the evolutionary retention of prion switching ability [7], [23], [32]. Even if one assumes very high rates of outcross mating [10], [11], these benefits are still significant in periods of environmental stress. Moreover, beneficial prion-based phenotypes can be “fixed” through repeated selection and meiotic recombination, allowing cells to lose any cost of the prion but maintain the beneficial trait [9], [16].

The distribution of fitness effects for genetic mutations is heavily skewed toward maladaptive phenotypes. Yet, the importance of mutation in shaping evolutionary change is not challenged [33]. Prions have now emerged as a paradigm-shifting mode of information transfer across generations, fueling debate over whether they, too, can have biological benefits. Because prion switching rates differ from DNA mutation rates by several orders of magnitude, these heritable elements provide an attractive and quantitatively distinct substrate for natural selection.

Of course, it is impossible to fully test if prions initially evolved explicitly to provide explicit benefits for adaptation or rather as parasitic elements that have subsequently been repurposed by their hosts. Mutations that produce disease can be maintained at high frequencies in natural populations if they also provide strong adaptive benefits (e.g., resistance to malaria from mutations that cause sickle cell anemia [34]). Likewise, “selfish” elements, such as plasmids, can act as evolutionarily useful conduits for information transfer, despite their known fitness costs. Whether prions represent a disease epidemic in fungi or a new layer of biological regulation based on “molecular memories” of past stressors will no doubt remain the subject of intense debate for years to come. However, the common presence of this mode of inheritance in natural yeast populations unequivocally establishes the importance of protein-based genes shaping the dynamics and evolution of microbial communities.
